# Covert oculo-manual coupling induced by visually guided saccades

**DOI:** 10.3389/fnhum.2013.00664

**Published:** 2013-10-10

**Authors:** Luca Falciati, Tiziana Gianesini, Claudio Maioli

**Affiliations:** Department of Clinical and Experimental Sciences and National Institute of Neuroscience, University of BresciaBrescia, Italy

**Keywords:** eye-hand coupling, saccade, transcranial magnetic stimulation, motor evoked potentials, motor excitability

## Abstract

Hand pointing to objects under visual guidance is one of the most common motor behaviors in everyday life. In natural conditions, gaze and arm movements are commonly aimed at the same target and the accuracy of both systems is considerably enhanced if eye and hand move together. Evidence supports the viewpoint that gaze and limb control systems are not independent but at least partially share a common neural controller. The aim of the present study was to verify whether a saccade execution induces excitability changes in the upper-limb corticospinal system (CSS), even in the absence of a manual response. This effect would provide evidence for the existence of a common drive for ocular and arm motor systems during fast aiming movements. Single-pulse TMS was applied to the left motor cortex of 19 subjects during a task involving visually guided saccades, and motor evoked potentials (MEPs) induced in hand and wrist muscles of the contralateral relaxed arm were recorded. Subjects had to make visually guided saccades to one of 6 positions along the horizontal meridian (±5°, ±10°, or ±15°). During each trial, TMS was randomly delivered at one of 3 different time delays: shortly after the end of the saccade or 300 or 540 ms after saccade onset. Fast eye movements toward a peripheral target were accompanied by changes in upper-limb CSS excitability. MEP amplitude was highest immediately after the end of the saccade and gradually decreased at longer TMS delays. In addition to the change in overall CSS excitability, MEPs were specifically modulated in different muscles, depending on the target position and the TMS delay. By applying a simple model of a manual pointing movement, we demonstrated that the observed changes in CSS excitability are compatible with the facilitation of an arm motor program for a movement aimed at the same target of the gaze. These results provide evidence in favor of the existence of a common drive for both eye and arm motor systems.

## Introduction

Reaching and manipulating objects under visual guidance is one of the most common motor behaviors in everyday life. Converging evidence from a large number of studies indicates that a tight coordination of gaze and arm movements is of paramount importance for the accurate execution of visually guided aiming tasks.

Typically, a saccade is made shortly before a hand movement is initiated (Prablanc et al., 1979; Lünenburger et al., 2000; Dean et al., 2011), thereby bringing the target into the fovea. If foveation of the target is prevented, pointing accuracy decreases considerably (Vercher et al., 1994; Henriques et al., 1998; Neggers and Bekkering, 1999; van Donkelaar and Staub, 2000; Medendorp and Crawford, 2002; Horstmann and Hoffmann, 2005). Moreover, subjects cannot fixate to a new target before the hand reach is completed, indicating that the gaze is anchored to the target during the entire pointing movement (Neggers and Bekkering, 2000, 2002). Finally, a high correlation between hand position and gaze error has been reported in pointing tasks to remembered visual targets (Flanders et al., 1999; Admiraal et al., 2003, 2004) or during an illusion in which the perceived direction of target motion is altered by a moving background (Soechting et al., 2001). Experimental evidence strongly supports the viewpoint that extraretinal gaze signals are required for precise pointing and reaching movements and that eye and arm motor control systems are mutually coupled (van Donkelaar and Staub, 2000; Engel and Soechting, 2003; Dean et al., 2011).

Some clinical observations also support the existence of a tight coupling between arm and eye control systems for fast aiming movements. Patients suffering from bilateral parietal lobe atrophy sometimes show a slavish dependence of reach on gaze (Buxbaum and Coslett, 1997; Carey et al., 1997). In fact, these patients are incapable of reaching objects they do not look at and inevitably move their hand to the place their eyes are fixating on (“magnetic misreaching”). A possible interpretation of these data is that whenever the eyes move, a motor plan is formed that carries the arm to the same target. Normally, eye or hand motor responses can be separately inhibited, depending on the ongoing behavioral goal. However, neurological disorders can induce a pathological incapacity to disjoin eye and upper-limb movements to the same visual target.

In a previous transcranial magnetic stimulation (TMS) study, we showed that smooth pursuit eye movements are linked to a modulation of corticospinal system (CSS) excitability of the resting arm (Maioli et al., 2007). Excitability changes were found to be compatible with a motor plan encoding an aiming movement of the hand toward the same target tracked by the eyes. In the presence of a common drive to ocular and arm motor systems, we expect that the execution of an eye saccade would also induce excitability changes in the upper-limb CSS in a pure oculomotor task. In this paper, we demonstrate that similar to smooth pursuit eye movements, highly specific changes in the upper-limb CSS excitability also occur in a strict temporal relationship with eye saccades, even if the task does not demand a manual response.

## Materials and methods

### Subjects

Nineteen adult volunteers (10 males and 9 females, mean age: 20.7 years, range: 20–23 years) with no history of head trauma or neurological disease participated in the study. All subjects were right-handed (as measured by the Edinburgh handedness inventory) and naïve to the purpose of the experiment. This study was conducted in accordance with the ethical guidelines set forth by the Declaration of Helsinki. Written informed consent was obtained from all participants.

### Eye movement and EMG recording

Horizontal and vertical eye movements were recorded (DC-200 Hz low-pass filtered) by means of electrooculography (EOG). Ag-AgCl electrodes were placed at the external canthi and above and below the right eye. EOG calibration was frequently repeated during the experimental session. Drift of DC offset was compensated within each trial by making the subject look at a central fixation cross before the presentation of the saccade target. Surface electromyograms (EMG) were recorded on the right hand side from the *first dorsal interosseous* (FDI), *abductor digiti minimi* (ADM), *extensor carpi radialis* (ECR), and *flexor carpi ulnaris* (FCU) muscles (1000× amplification; 0.2 Hz −1 kHz bandwidth). EOG and EMG signals were digitally converted at a sampling rate of 5 kHz (National Instruments PCI-MIO-16E-4) and analyzed off-line using custom-written Labview software (National Instruments, Austin, TX).

### Transcranial magnetic stimulation

A 70 mm figure-of-eight double coil connected to a MagStim Super Rapid magnetic stimulator (Mag-1450-00, MagStim Co. Ltd Whitland, UK) was positioned over the left motor cortex, contralateral to the EMG recorded muscles. The coil was placed tangentially to the scalp with the handle pointing backwards and laterally at a 45° angle to the sagittal plane. This orientation was chosen because the lowest motor threshold is achieved when the induced electrical current in the brain flows approximately perpendicular to the central sulcus (Mills and Nithi, 1997).

The scalp site at which motor evoked potentials (MEPs) were elicited at the lowest stimulus strength in the FDI muscle was determined. Once the optimal scalp site was found, the coil was securely fixed in place with an appropriate mechanical device. The response threshold was defined as the stimulus intensity at which 5 out of 10 consecutive stimuli at the optimal site evoked an MEP with a peak-to-peak amplitude of at least 100 μV in the relaxed muscle. Stimulus intensity during the entire stimulation paradigm was set at 1.2 times the FDI motor threshold. The mean stimulation intensity across subjects was equal to 71.5% of the maximum power of the magnetic stimulator. This stimulation intensity at the optimal scalp site for FDI also evoked MEPs in the ADM, ECR, and FCU muscles in almost all experimental sessions, although these MEPs generally occurred at a considerably lower amplitude. In order to ensure that excitability changes were measured against a reliable baseline for each muscle, subjects were included in the analysis only if the evoked responses were larger than the above defined threshold amplitude. This acceptance criterion was fulfilled in 15, 18, and 18 subjects for the ADM, ECR, and FCU muscles, respectively. Furthermore, 2 subjects had to be excluded from the analysis of the ADM muscle due to technical issues in recording.

### Experimental protocol

**Figure 1:**
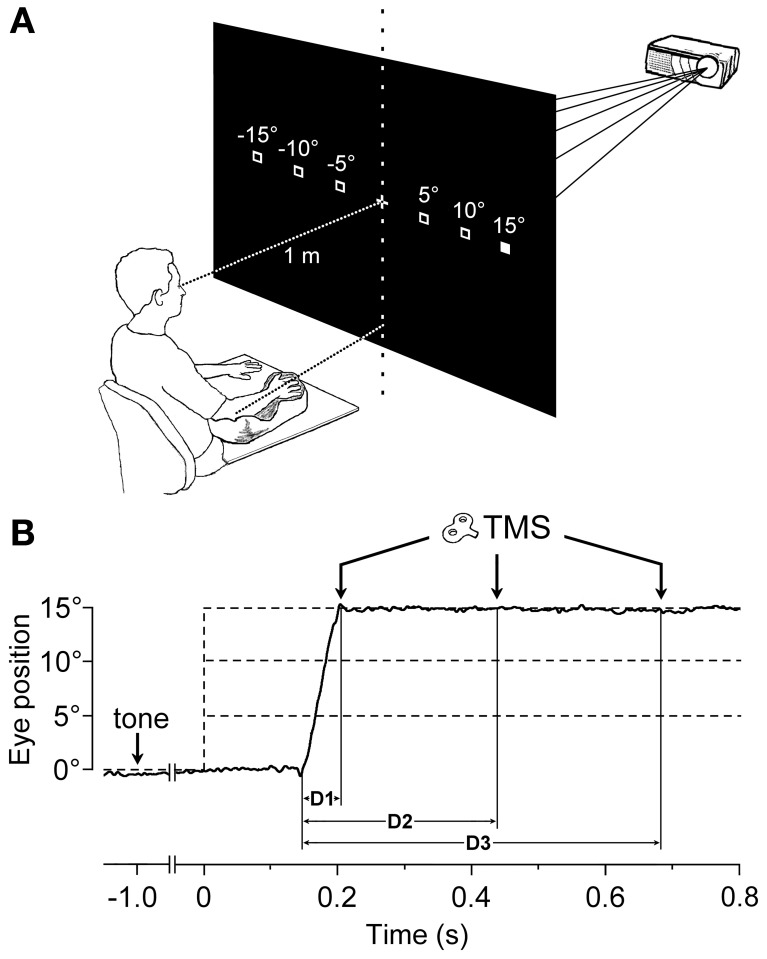
**Experimental Setup and Protocol. (A)** The target stimulus was projected at one of six possible spatial locations. The filled square represents the actual visual target, while empty squares indicate the other possible positions. The head was immobilized using a chin-rest and a head-support device (not shown). Notice the arm-hand posture imposed on the subject with respect to the central vertical meridian (see text). **(B)** An EOG recording (solid line) during a representative saccadic eye movement executed toward a 15° eccentric target. Dashed lines indicate the onset (vertical) and the possible eccentricities (horizontal) of the visual target, which was presented 1000 ms after a warning tone. TMS pulses were randomly delivered at 60 ms (D1), 300 ms (D2), or 540 ms (D3) after the beginning of the saccade.

Subjects sat comfortably, with their right upper-limb resting in a relaxed position on a horizontal support. The support was formed by a polystyrene-bead vacuum splint and molded on the hand palm and forearm of the subject. This device enabled the limb to be loosely restrained in order to maintain the longitudinal axis of the pronated hand horizontally aligned with the forearm and pointing toward the central vertical meridian (Figure 1A). The head was stabilized using a combination chin-rest and head-support device. White visual stimuli were rear-projected on a wide tangent black screen (160 cm in width and 120 cm in height) that was placed 1 m in front of the subject. Participants had to fixate on a central cross for 3 s. One second after a warning tone, the fixation cross turned off, and a new target (a square subtending 0.6° of visual angle) randomly appeared for 3 s at one of 6 peripheral positions along the horizontal meridian, at eccentricities of 5°, 10°, or 15°, in the left or right visual field. Subjects were instructed to quickly respond by moving their gaze to the peripheral stimulus, and to fixate it as accurately as possible. After the target turned off, the central fixation cross was presented again, marking the beginning of a new trial.

Particular attention was paid during task explanation to avoid drawing the subject's attention to the possibility of making an aiming arm movement toward the target. In addition, the lack of any imagery of manual pointing movements was assessed through a subject interview after the experimental session. In order for a trial to be included in the analysis, the following criteria had to be fulfilled: (1) subjects had to keep their muscles completely relaxed, as defined by the absence of any detectable EMG activity for the entire task duration; (2) the latency of the saccadic response had to be shorter than 300 ms in order to ensure the presence of a high and constant arousal level in the subject; and (3) the saccadic responses could not be anticipatory. To this end, trials with a saccadic latency shorter than 90 ms were discarded.

During each trial, single-pulse TMS was triggered by the EOG signal at one of 3 different time delays, with respect to the saccade onset (Figure 1B). Labview software controlling the experiment was programmed in order to randomly deliver TMS pulses at 0, 250, or 500 ms after saccade onset, as identified by a real-time analysis of the EOG signal. However, the buffered data acquisition (with buffer time epochs of 40 ms) imposed a certain degree of uncertainty to the recognition of saccadic occurrence. Therefore, actual TMS delays with respect to the precise saccade onset were computed off-line, trial-by-trial, using a semi-automatic computer-based analysis. The mean values (±SD) of TMS latencies with respect to saccade onset for the 3 delay conditions were as follows: 56 ± 15 ms (shortly after the end of saccade execution), 296 ± 19 ms, and 537 ± 23 ms. For the remainder of this report, these 3 delay conditions will be conventionally referred to as *D*1 = 60 ms, *D*2 = 300 ms, and *D*3 = 540 ms.

Each experimental session comprised 5 blocks of 36 trials (with 3 min intervals between blocks), yielding an overall number of 180 trials (10 trials for each of the 18 target-eccentricity × TMS-delay conditions).

### Statistical analysis

MEP amplitudes are continuous variates characterized by a very large variability among subjects in the mean and standard deviation of their statistical distribution. Therefore, in order to compare data from different subjects and apply statistical significance tests, data transformation was required. Details on the adopted statistical procedures have been previously described (Maioli et al., 2007). Briefly, for each subject, raw data were transformed as follows:

$$X=\frac{x-\mu_{s}}{\sigma_{s}}$$

where *X* is the standardized measure and *x* is the original experimental value having a distribution with μ_s_ mean and σ_*s*_ standard deviation. This transformation normalized the experimental variability and permitted direct data comparison among subjects because MEP amplitude became a standard variate with zero mean and unitary standard deviation. For each subject, μ_*s*_ was obtained by averaging peak-to-peak MEP amplitudes across all experimental conditions. The standard deviation σ_*s*_ was computed as the square-root of the residual variance within each subject, i.e., the variance within groups defined by all combinations of target-eccentricity and TMS-delay conditions. Using this procedure, the intra-subject variability of experimental data was normalized without cancelling the effects that might result from the application of the different experimental conditions.

In order to test the statistical significance of the effects of target position on MEP amplitude, a Two-Way ANOVA for repeated measurements was performed on the average MEP amplitudes computed for each subject for a given experimental condition. The factors were “*side*” (left vs. right visual hemifield) and “*eccentricity*” (5°, 10°, 15°). Separate analyses were run for each muscle and TMS delay.

## Results

### Overall changes of CSS excitability in upper-limb muscles

**Figure 2:**
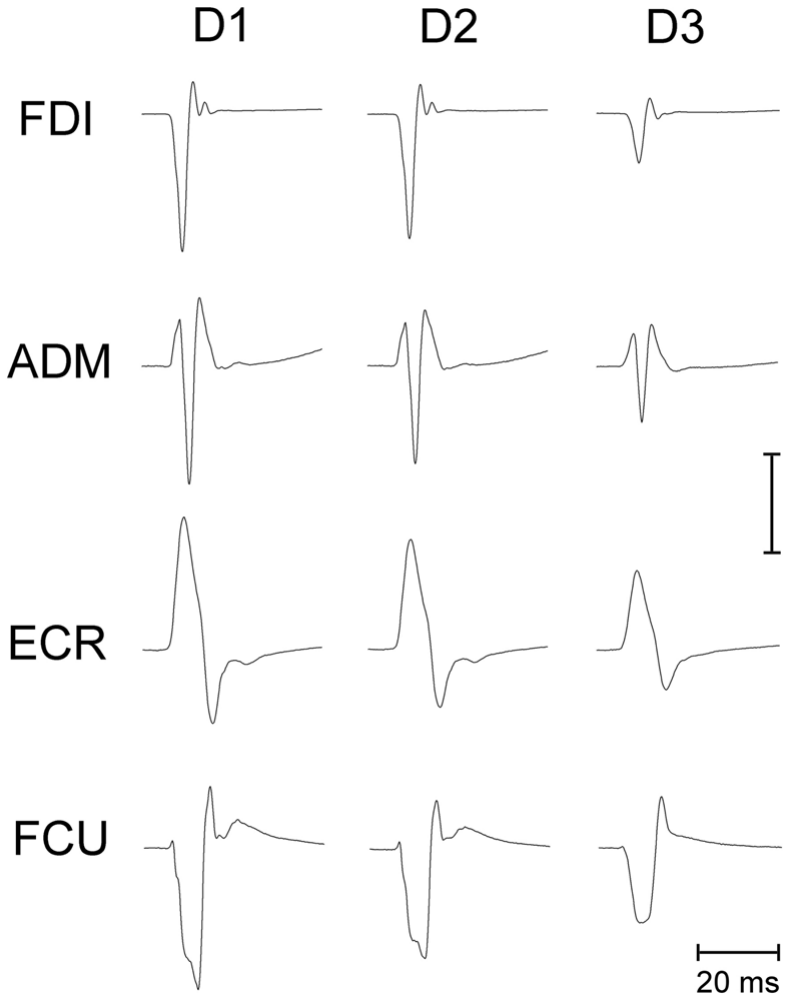
**Average EMG recordings from relaxed FDI, ADM, ECR, and FCU muscles in a representative subject**. All muscles show a progressive decay of MEP amplitude as a function of the TMS delay (D1, D2, and D3) from saccade onset. The amplitude calibration mark represents 1.0, 0.2, 0.35, and 0.2 mV for the FDI, ADM, ECR, and FCU muscles, respectively.

Making a saccadic eye movement to a peripheral target induced clear-cut excitability changes in the CSS of the resting arm, which were strictly time-locked to saccade execution. The mean saccadic latency across subjects recorded within our experimental condition was 158 ± 34 ms. Figure 2 shows the mean MEPs recorded from the 4 investigated muscles in a representative subject as a function of the TMS delay from saccadic onset and irrespective of the target position. Each trace represents the average MEP of 54–59 TMS stimuli delivered in a single experimental session. Trials with different TMS delay values were presented in a random order so that subjects were completely unaware of the timing of the TMS occurrence. It is clear that MEP amplitude is largest at *D1* (~60 ms after saccade onset) and gradually decreases at longer TMS delays.

**Figure 3:**
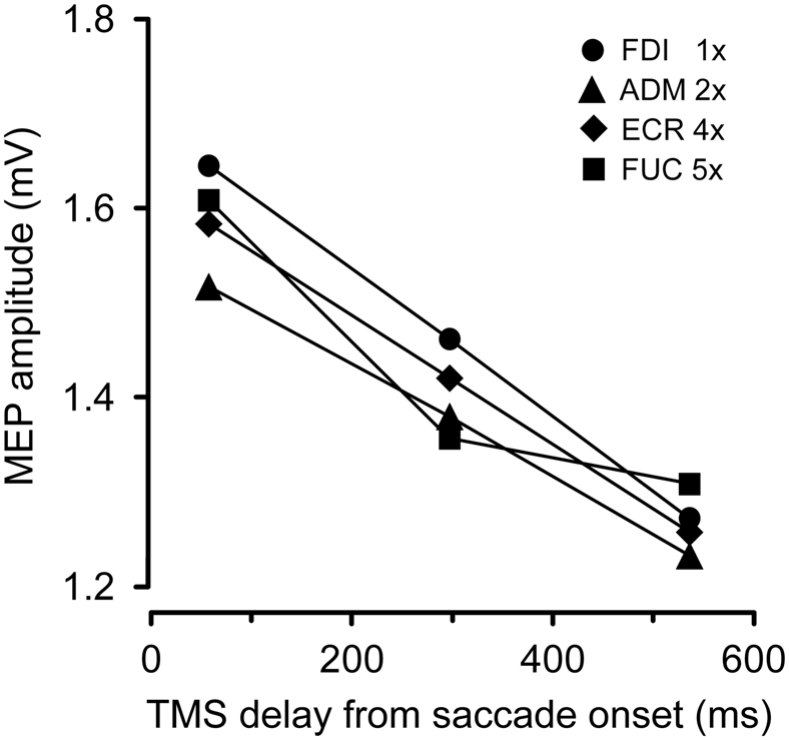
**Changes in mean MEP amplitudes as a function of TMS delay from saccade onset for each recorded muscle**. Depicted MEP amplitudes represent the grand mean across subjects and target positions. MEP amplitudes for different muscles have been scaled according to the values shown in the legend.

Figure 3 depicts the average variation across subjects in peak-to-peak MEP amplitude as a function of the TMS delay and irrespective of the target position. The CSS excitability decays linearly in all muscles. On average, mean MEP amplitude at TMS delay *D3* is ~20% smaller than shortly after saccade onset.

Our experimental protocol did not allow a direct measurement of resting MEP amplitudes, i.e., during fixation before the saccade onset. Therefore, we cannot determine whether this decay is the result of a sudden transient increase of excitability occurring before or in correspondence to the execution of a saccade or whether it reflects a progressive CSS depression lasting for several hundreds of milliseconds following the onset of a saccadic eye movement.

### Effects of side and eccentricity of the saccadic target on excitability changes

**Figure 4:**
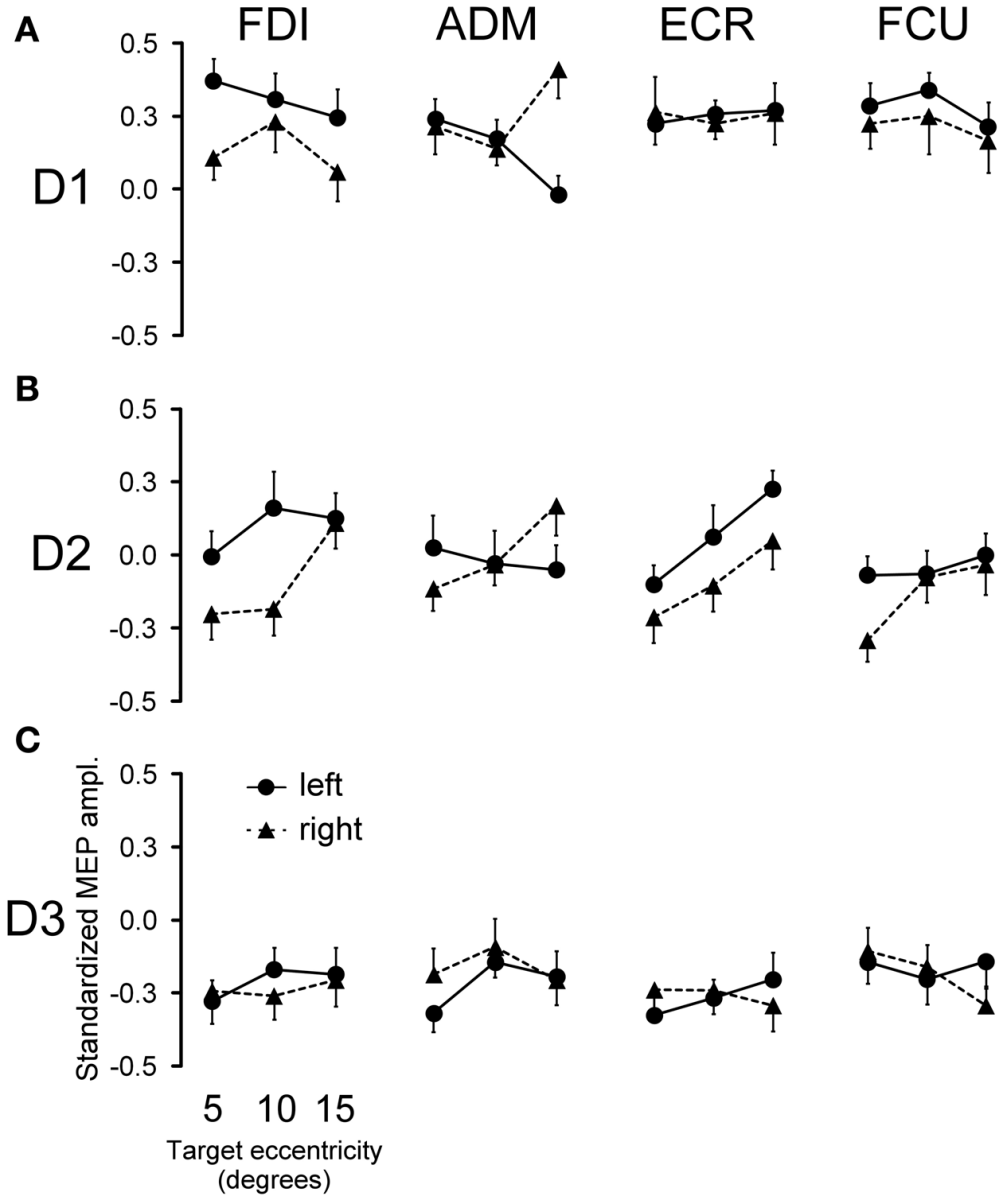
**Effects of saccadic target position on MEP amplitude**. The modulation of mean MEP amplitude across all subjects following saccade execution is shown for each muscle. Points represent the mean standardized MEP amplitudes as a function of target eccentricity and visual hemi-field of presentation for TMS delay D1 **(A)**, D2 **(B)**, and D3 **(C)**. Error bars represent the standard error of the mean.

In addition to the decay in overall CSS excitability, MEP amplitudes are modulated in a highly specific manner in different muscles, depending on the target position of the visually guided saccade and on the timing of TMS stimulation with respect to saccade onset. Figure 4 shows the average values across subjects of standardized MEP amplitudes for all recorded muscles as a function of TMS delay, visual hemi-field of target appearance and target eccentricity.

No changes in mean MEP amplitude occurred at TMS delay *D3* as a function of target position (Figure 4C). By contrast, quite large MEP modulations were observed in some muscles at *D1* and *D2*, depending on both the direction and eccentricity of the visually guided saccade (Figures 4A,B).

In order to test the statistical significance of the observed changes in motor excitability, a Two-Way repeated measures ANOVA (Table 1) was performed for each muscle and TMS delay on the mean MEP amplitudes of each subject, with the target side and eccentricity as grouping factors. The analysis at delay *D1* demonstrates that the direction of the saccadic movement (“*side*” factor) had a significant principal effect on MEP amplitude only for the FDI and ADM muscles. Furthermore, in the ADM muscle, a significant “*eccentricity* × *side*” interaction was also present. Figure 4A shows that the “*side*” principal effect in the FDI muscle was determined by the fact that MEP amplitude after leftward saccades was considerably larger than after rightward saccades, independently of the target eccentricity. By contrast, an opposite modulation of MEP amplitude as a function of saccade direction was observed in the ADM muscle, which increased in size at the most eccentric target position of 15° [*F*_(1, 14)_ = 11.667, *P* = 0.004]. Conversely, no significant changes in MEP amplitude were observed as a function of either side or eccentricity of the target in the more proximal ECR and FCU muscles.

**Table 1:** **Two-Way repeated measures ANOVAs of MEP amplitudes**.

**Muscle (N)**	**Eccentricity**		**Side**		**Ecc. × Side**	
	***F*_2,_ (*N*− 1) × 5**	***P***	***F*_1_, (*N*− 1) × 5**	***P***	***F*_2_, (*N* − 1) × 5**	***P***
**D1 60 ms**						
FDI (19)	1.148	0.322	**7.158**	0.009^**^	0.677	0.510
ADM (15)	0.528	0.592	**4.589**	0.036^**^	**7.159**	0.001^**^
ECR (18)	0.051	0.950	0.000	0.994	0.098	0.907
FCU (18)	0.703	0.498	0.831	0.364	0.027	0.973
**D2 300 ms**						
FDI (19)	2.732	0.071	**5.771**	0.018^**^	1.507	0.227
ADM (15)	0.672	0.514	0.095	0.758	1.722	0.186
ECR (18)	**5.998**	0.004^**^	**4.815**	0.031^**^	0.087	0.917
FCU (18)	1.999	0.142	1.740	0.191	0.970	0.383
**D3 540 ms**						
FDI (19)	0.332	0.719	0.146	0.704	0.290	0.749
ADM (15)	1.429	0.246	0.773	0.382	0.413	0.663
ECR (18)	0.121	0.886	0.018	0.892	0.709	0.495
FCU (18)	0.809	0.449	0.160	0.690	1.138	0.325

N is the number of subjects.

** denotes statistical significance (P < 0.05).

A different picture emerges from the analysis of the data at the intermediate TMS delay *D2*. Both “*eccentricity*” and “*side*” of the saccadic target induced a clear-cut modulation of MEP amplitude in the ECR muscle. By contrast, only a “*side*” principal effect was found in the FDI muscle. No statistically significant effects were detected on MEPs recorded in the ADM and FCU muscles. It should also be noted that in all muscles, “*eccentricity*” and “*side*” factors did not have a statistically significant interaction.

Finally, at the longest delay of TMS pulse delivery (~540 ms after saccade onset), the target position of visually guided saccades was completely ineffective at inducing changes of motor excitability in all muscles (delay *D3* in Table 1 and Figure 4C). This last finding supports the conclusion that the above described target-dependent modulations of CSS excitability in upper-limb muscles are highly specific and strictly time-locked to saccade execution.

**Figure 5:**
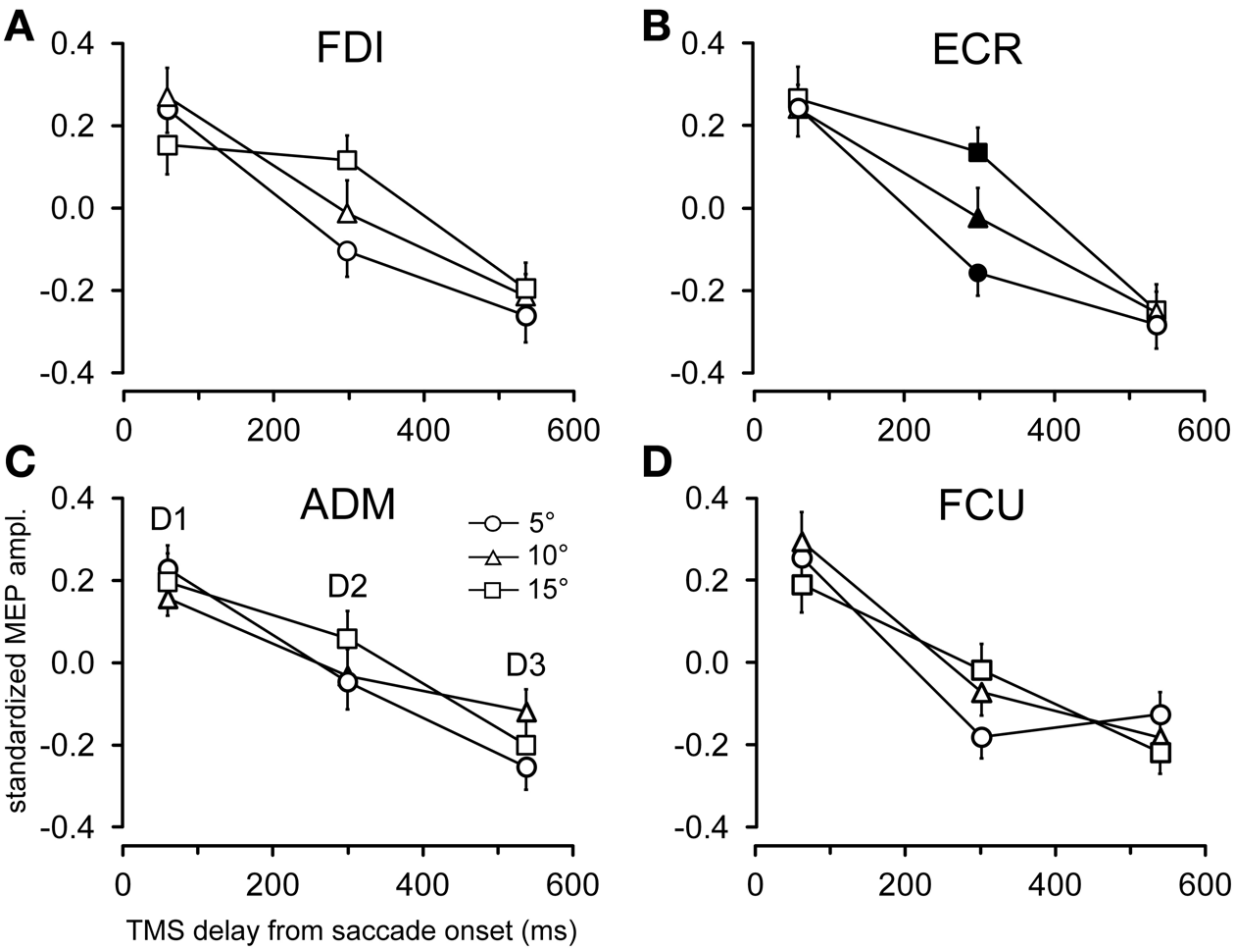
**Effects of target eccentricity on MEP amplitude**. Mean MEP amplitudes across subjects are shown as a function of TMS delay for target eccentricities of 5°, 10°, and 15°, irrespective of the visual hemi-field of stimulus presentation, in the FDI **(A)**, ECR **(B)**, ADM **(C)**, and FCU **(D)** muscles. Filled symbols denote a statistically significant difference between MEP amplitudes at a given TMS delay. Error bars represent the standard error of the mean.

**Figure 6:**
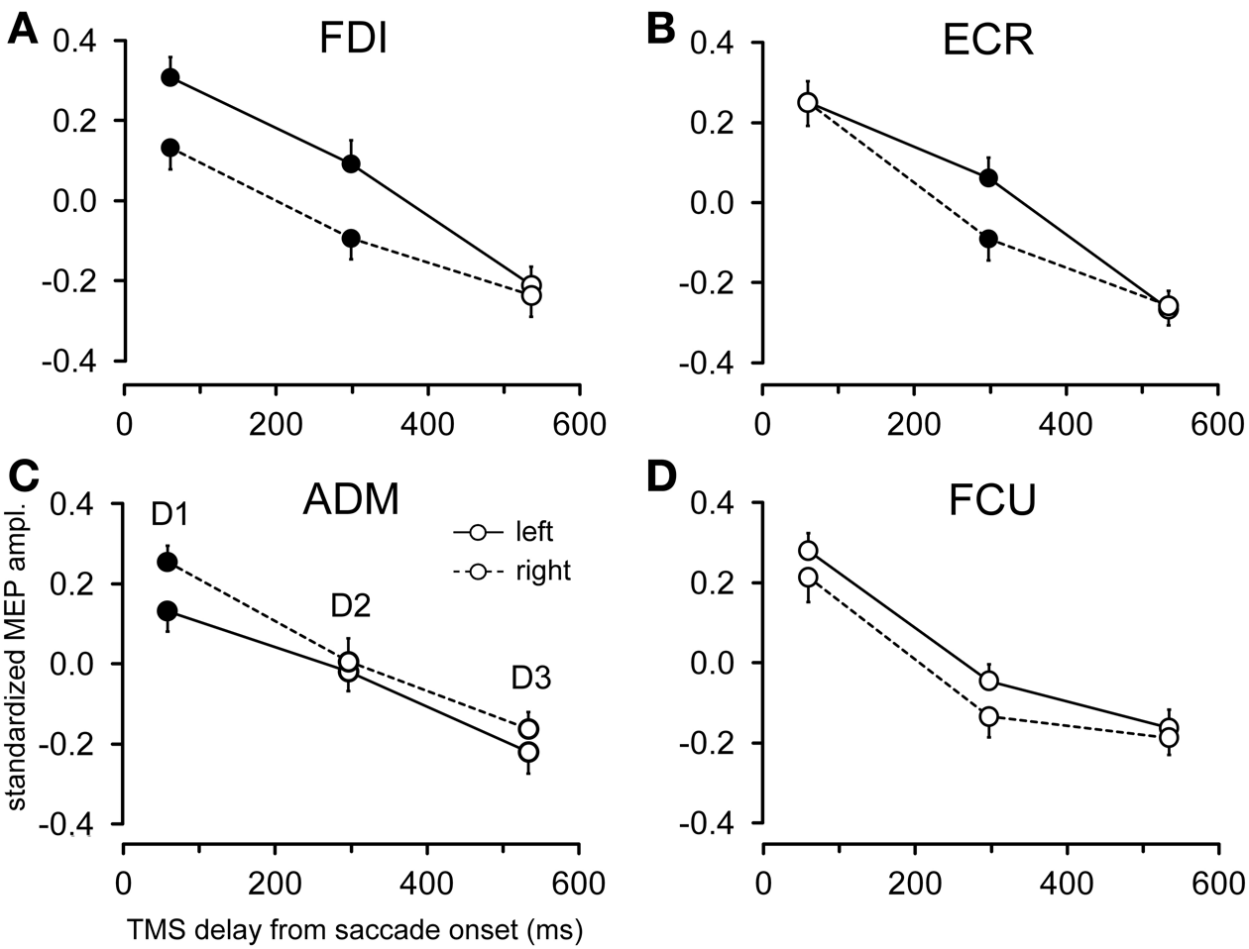
**Effects of target side on MEP amplitude**. Mean MEP amplitudes across subjects are shown as a function of TMS delay for leftward and rightward saccades, irrespective of target eccentricity, in the FDI **(A)**, ECR **(B)**, ADM **(C)**, and FCU **(D)** muscles. Filled symbols denote a statistically significant difference between MEP amplitudes at a given TMS delay. Error bars represent the standard error of the mean.

The time course of the effects of target position on CSS excitability can be better appreciated in Figures 5, 6, where the influences of target eccentricity and side on the mean MEP amplitude are separately plotted for each muscle. This procedure is justified by the lack of a significant “*eccentricity* × *side*” interaction in the ANOVAs (except at delay *D1* in the ADM muscle). Figure 5 illustrates the changes in motor excitability induced by target eccentricity. Filled symbols indicate that the differences in MEP amplitude at a given TMS delay were statistically significant. The graphs demonstrate that, on top of the overall decay in MEP amplitude, a time-locked specific modulation of motor excitability as a function of saccade amplitude occurred in the ECR muscle (Figure 5B) ~300 ms after saccade onset. A similar trend can also be observed for the FDI muscle (Figure 5A), but this effect did not reach statistical significance. By contrast, the excitability of the ADM and FCU muscles (Figures 5C,D) steadily decreased after saccadic execution but did not show any sign of a specific modulation as a function of saccade amplitude. The excitability decay also appeared somewhat faster in the FCU muscle than in the other muscles (Figure 5D).

Figure 6 shows the changes in motor excitability related to the side of target appearance (same conventions as in Figure 5). Here too, a specific modulation of MEP amplitude depending on the direction of the eye movement occurred in a precise time window after saccade onset; this effect was absent at TMS delay *D3*. In contrast to the target eccentricity, a side-specific modulation of MEP amplitude was present, although on the opposite sign in both the FDI and ADM muscles; this modulation occurred early after saccade onset at TMS delay *D1* (Figures 6A,C). Furthermore, a statistically significant difference in motor excitability was also observed in the FDI and ECR muscles at delay *D2* (Figures 6A,B), with MEP amplitudes larger in both muscles after leftward compared to rightward saccades.

## Discussion

The hand rarely moves without being coupled to an eye saccade. Although saccades can be performed without any accompanying limb movement, they are often functional as a means to gather visuospatial information for motor coordination while reaching or manipulating objects. Our results clearly demonstrate that the execution of visually guided saccades involves excitability changes in the motor control system of the arm, in the absence of any overt upper-limb movement or sign of EMG activation. These changes last for at least 300 ms after eye movement onset and reveal an overall decay in excitability of the upper-limb CSS, which is modulated in a highly specific manner in hand and wrist muscles, depending on saccade direction and target eccentricity. Therefore, this effect cannot be generically ascribed to arousal level or to a non-specific variation of cortical excitability resulting from task execution. Similar to what was previously described in a smooth pursuit task (Maioli et al., 2007), we believe that the observed changes in CSS excitability are compatible with the facilitation of a motor program for an upper-limb movement aiming at the same target of the gaze. One may argue that the changes in excitability of the motor cortex in this study could be caused by mental imagery of the pointing arm movement by the subject. However, this explanation seems unlikely because we carefully avoided drawing participants' attention to the possibility of making an aiming arm movement. Moreover, interviews after the experimental session confirmed the absolute absence of any imagery of manual pointing.

Specific modulations in MEP amplitude are inscribed on a global linear decay in CSS excitability, whereby responses in all muscles are smallest at the longest tested TMS delay after saccade onset. Our paradigm does not allow us to ascertain whether this excitability decay is the result of an initial facilitation that slowly decreases toward baseline values or represents a progressive CSS inhibition following saccade execution. However, it is interesting to notice that in a previous smooth pursuit study (Maioli et al., 2007), gaze movements were linked to an overall decrease in excitability of the motor control system of the resting arm. This finding was interpreted as a mechanism to prevent muscle contraction in an eye tracking task that engages the upper-limb motor system but does not require an overt manual response.

On top of the decay in overall excitability, changes in MEP amplitude present a complex time-dependent modulation as a function of the direction and/or amplitude of the saccadic movement, whose interpretation requires a separate discussion for each TMS delay. Data show that the modulation of MEP amplitudes in relation to target position extinguishes at TMS delay *D3*, i.e., ~540 ms after saccadic onset. It is interesting to compare this finding with the temporal coupling between eye and hand movements in an oculo-manual pointing task. Sailer et al. (2000), using visual stimuli presented with a procedure almost identical to our experimental method, reported that the hand arrives at the target in less than 500 ms after the beginning of the eye movement, with a saccade latency very similar to that found in our study. Under the hypothesis that the observed modulations in MEP amplitude reflected the activation of a sub-threshold motor program for an aiming hand movement, we should expect that TMS at delay *D3* would test upper-limb CSS excitability beyond the time interval within which a hand pointing movement is normally executed. Accordingly, no changes in MEP amplitude are observed as a function of target position. Furthermore, eye-hand coordination studies in aiming manual tasks have repeatedly reported that saccadic movement starts between 70 and 90 ms before the initiation of the hand movement (Carnahan and Marteniuk, 1994; Helsen et al., 1998; Lünenburger et al., 2000; Sailer et al., 2000). This means that at delay *D1* (60 ms), TMS is delivered shortly before the expected time of the hand movement onset during an eye-hand coordination task.

At TMS delay *D1*, when the overall CSS excitability is at its highest level, MEP amplitude modulation related to saccade direction is only limited to the most distal muscles. The FDI muscle (abductor of the index finger) on the right-hand side shows higher MEP amplitudes after leftward compared to rightward saccades. The activation of this muscle with a pronated hand posture would indeed produce a leftward deviation of the index finger, i.e., in the same direction of the saccadic eye movement. Therefore, this MEP modulation is compatible with a coarse sub-threshold finger motor program coupled with gaze direction, as CSS excitability modulation does not scale with saccade amplitude. Similarly, the ADM muscle shows a side-specific modulation, which becomes more evident at the largest saccade amplitudes (15°). Interestingly, the excitability changes are opposite to that observed in the FDI muscle, i.e., MEP amplitudes are largest following rightward saccades. ADM muscle activation in a pronated hand would then induce a rightward deviation of the little finger. Therefore, also in this case we observe a facilitation of a finger movement that is congruent with the direction of the preceding eye saccade.

**Figure 7:**
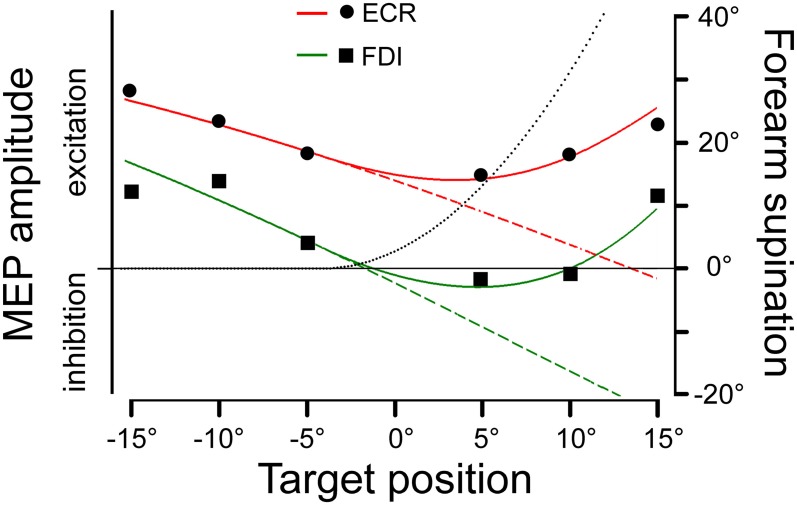
**Model predictions of changes in MEP amplitude in the ECR and FDI muscles as a function of saccadic target position**. Experimental mean MEP amplitudes in arbitrary units are depicted by dots and squares for the ECR and FDI muscles, respectively. Model predictions are shown by red (ECR) and green (FDI) traces and by varying (continuous lines) or maintaining constant (dashed lines) the angle of forearm supination. The black dotted line illustrates the changes in the forearm supination angle, which predicted the best fitting of the experimental data.

MEP amplitude modulations are much more difficult to interpret at TMS delay *D2*, which corresponds to ~300 ms after saccade onset. Here, CSS excitability varies with target position only in the FDI and ECR muscles. However, the relationship cannot be interpreted in a straightforward manner. Figure 7 shows the mean amplitudes of MEPs recorded in the FDI (squares) and ECR (dots) muscles as a function of saccadic target position. Negative values in the abscissa indicate a target location in the left visual field. Plotted data are those of Figure 4B but were scaled to arbitrary units in order to provide the best-fit with the predictions of the model, as presented in the next section. For both muscles, CSS excitability decreases from –15° to +5° target positions, but thereafter it increases again at the most eccentric positions in the right visual field. In fact, at a target eccentricity of 15°, mean MEP amplitudes did not show a statistically significant difference between the two visual hemi-fields.

### Modeling saccade-related modulation of MEP amplitude

In an attempt to find a functional interpretation of these modulations in CSS excitability, we tried to model the observed changes in MEP amplitude under the assumption that they reflect a facilitation of an aiming movement of the upper-limb toward the same target of the eye saccade. Furthermore, the model was construed by taking into account the undeniable fact that, if the hand begins to move from a fully pronated posture (Figure 1A), a natural pointing movement of the right limb toward a target in the right visual field involves a certain degree of forearm supination.

**Figure 8:**
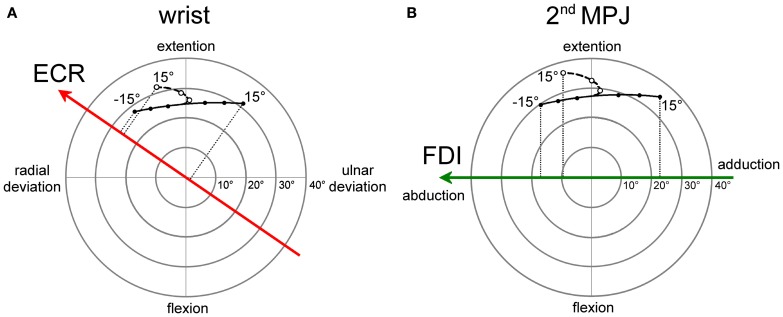
**Polar plot representations of the angular motion of wrist (A) and 2nd metacarpo-phalangeal (B) joints that were utilized for computing model predictions**. Bold vectors represent the pulling directions of the ECR (red) and FDI (green) muscles in the respective joint angular space. The ECR **(A)** and FDI **(B)** muscles are activated in a single-joint pointing movement by an amount proportional to the orthogonal projection of the movement representation in the joint angular space onto the muscle pulling vector. Filled dots connected by a continuous line describe single-joint pointing movements in the absence of changes in forearm supination. Empty dots connected by a dashed line describe pointing movements occurring in the presence of a variable amount of forearm supination computed by the model-fitting procedure.

The angular motion of the wrist and of the 2nd metacarpo-phalangeal joints (MPJ) can be vectorially represented in polar plots, as depicted in Figures 8A,B respectively. Each joint has two degrees-of-freedom, corresponding to flexion/extension and radial/ulnar deviations for the wrist and to flexion/extension and abduction/adduction for the 2nd MPJ. The pulling directions of the ECR and FDI muscles in the respective joint angular space are shown by the bold oriented axes. The ECR pulling direction (Figure 8A) was computed by vectorial summation of the maximal isometric force vectors reported by gaard and Walsh (Bawa et al. 2000) for the *longus* and *brevis* heads of the muscle. It should be noticed that the ECR muscle is not a pure extensor, but its action is even stronger in producing a radial deviation of the wrist. By contrast, the FDI muscle can be considered as a pure abductor of the index finger (Figure 8B).

Filled dots connected by a continuous line in the polar plots represent the angular deviation that should be made in a single-joint movement to direct the hand (Figure 8A) or the index finger (Figure 8B) toward the visual target of the eye saccade. Of course, a real manual aiming movement would involve several joints of the upper-limb at the same time. Therefore, the actual activation of each muscle would be determined in a complex manner by the multi-jointed movement of the whole limb. However, as a rough approximation, it is fair to assume that a motor program for an aiming movement would activate the ECR and FDI muscles by an amount proportional to the orthogonal projection (dotted lines in Figures 8A,B) of the movement vector in the single-joint angular space onto the oriented axis representing the muscle pulling direction. If the projection vector has the same sense as the pulling direction vector, we should expect facilitation in muscle excitability; if the two vectors have an opposite sense, the muscle should be inhibited. This theoretical muscle activation as a function of target position is represented by dashed lines in Figure 7 for the ECR (red) and FDI (green) muscles. Clearly, the experimental data points diverge from this prediction. In fact, when visually guided saccades are directed to the right hemi-field, MEP amplitudes of both the ECR and FDI muscles are expected to decrease monotonically with target eccentricity, while experimentally they start to increase again at eccentricities larger than 5°.

As mentioned above, a more realistic model should, however, take into account that if the forearm starts in a fully pronated posture, an aiming movement to a visual target on the right hemifield would involve a certain degree of hand supination. If the forearm rotates about its long axis, then the movement vector in the intrinsic joint angular space rotates to the opposite direction, as shown by the dashed trajectories and empty dots in the polar plots of Figure 8 for both the wrist (**A**) and 2nd MPJ (**B**). The black dotted line in Figure 7 shows the forearm supination angle that should be associated with the aiming movement to provide the best least-squares fit with experimental MEP data from both muscles. For sake of simplicity, the fitted function describing the change in the supination angle was a second-order parametric curve. The only constraint imposed to the fitting algorithm was that a fully pronated position (0°) of the forearm had to be maintained at the most eccentric target positions on the left visual hemifield. The parameters of the second-order curve and the target position at which hand supination began to develop were computed by the non-linear fitting procedure. Fitted parameters reasonably predicted that the supination of the forearm from its initial pronated posture would begin with a target position at a few degrees to the left of the central fixation point and would increase to approximately 50° for a target eccentricity of 15° in the right hemi-field. When a certain degree of supination is taken into account, model predictions fit very well with the experimentally observed changes of MEP amplitude in both the ECR and FDI muscles. It should also be stressed that the proposed model does not intend to describe the entire motor program of a hand pointing movement coupled with the eye saccade. Instead, the model attempts to predict muscle activation during a possible manual pointing movement toward the gaze target, coherent with the changes in CSS excitability photographed by TMS in a narrow time interval at about 300 ms after saccadic onset.

In summary, this very simplistic model with few reasonable assumptions demonstrates that the experimentally observed changes in MEP amplitude are compatible with the facilitation of a motor program, whose goal is to direct the hand toward the same target as that of the gaze movement. Although other functional interpretations of the experimental data can be conceived, our model provides a suitable conceptual framework for the clear fact that within a narrow time window after the occurrence of a visually guided saccade, the excitability of the CSS controlling upper-limb musculature undergoes highly specific modulations, which are tightly correlated with the target position of the preceding gaze movement.

### Behavioral and neurophysiologic evidence of a common drive for eye and hand movements

In natural tasks, eye and arm movements are tightly linked (Neggers and Bekkering, 2000, 2002). It is well-known that eyes begin to move and arrive at the target before the hand (Prablanc et al., 1979; Lünenburger et al., 2000; Sailer et al., 2000; Dean et al., 2011), resulting in more accurate manual pointing compared with when the hand moves alone (Vercher et al., 1994; Henriques et al., 1998; Neggers and Bekkering, 1999; van Donkelaar and Staub, 2000; Medendorp and Crawford, 2002; Horstmann and Hoffmann, 2005). It should also be noted that short-term adaptation of saccadic gaze amplitude induces congruent changes also in the amplitude of goal-directed limb movements (Kröller et al., 1999), indicating that plastic changes must occur in a common neural substrate. Moreover, studying eye movements in task-oriented behaviors has demonstrated that saccades are not normally directed to the most visually salient points of the visual scene, but rather to objects or locations that are relevant for the task to be executed (Land et al., 1999; Hayhoe et al., 2003; Hayhoe and Ballard, 2005). In particular, gaze is most often directed toward the point of contact of objects that will subsequently be the target of a reach or toward critical points that are highly relevant for guiding the ongoing motor act (Johansson et al., 2001; Hayhoe et al., 2003).

The large amount of behavioral evidence in favor of a tight eye-hand coupling has stimulated the proposal of several models capable of simulating temporal and spatial properties of oculo-manual coordination. Two main approaches have been used to explain eye-hand coupling: (1) synchronization as a consequence of a common command to separate control systems for the two effectors (e.g., Howard, 1971; Bock, 1987) and (2) mutually coupled controllers exchanging information during movement, whereby coordination is ultimately achieved (e.g., Lazzari et al., 1997; Dean et al., 2011). Model predictions of reaction time correlations during eye-hand coordination in monkeys have been shown to be compatible only with a mutual excitation between two effector-specific controllers, but not with the common input hypothesis (Dean et al., 2011).

The existence of a saccade-related modulation of upper-limb CSS excitability, which is compatible with a sub-threshold motor plan for an aiming hand movement even in a pure oculomotor task, strongly supports the hypothesis that saccadic and manual control systems share a common input signal. This conclusion is corroborated by a similar modulation of upper-limb CSS excitability described during smooth pursuit eye movements (Maioli et al., 2007). This viewpoint is also supported by the finding of a shared internal representation of end position for both eye and arm movements and by the fact that both effectors always move to the same target when multiple targets are present (Gielen et al., 1984). Spatial coupling and our TMS data can hardly be accounted for without assuming the presence of a common command signal, at least at the early stages of sensorimotor integration and/or at the level of the visual signal processing mechanism. It should also be noted that common command signals and information exchange between effector-specific controllers are not mutually exclusive mechanisms for explaining eye-hand coordination.

The idea that eye movements are linked to a plan for an aiming movement of the arm to the same target also is supported by a particular form of optic ataxia, in which misreaching occurs only when targets are presented in non-foveal vision. Some patients with a lesion in the posterior parietal lobe show a slavish dependence of reach on gaze (“magnetic misreaching,” Buxbaum and Coslett, 1997; Carey et al., 1997). In fact, they inevitably move their hand to the spatial target they are fixating on, being incapable of reaching objects that they are not looking at. However, their performance is normal when allowed to foveate on the target. Because we found that a motor plan for the hand is normally associated with saccadic eye movements, this neurological defect could be interpreted as an incapacity to suppress the arm motor program when a hand movement is not required. This neurological defect may be due to a misfunctioning of the parietofrontal cortical network that underlies the control of visually guided reaching behavior. A common control mechanism at the early stages of sensorimotor integration for pointing and saccades is also supported by the neurophysiologic finding of a lack of strictly effector-specific visuospatial maps in frontal and parietal cortical areas. In fact, various fMRI studies in humans (Hagler et al., 2007; Levy et al., 2007; Beurze et al., 2009) have shown a large overlap in the neural circuitry involved in the preparation of saccades and hand pointing movements, including the frontal eye fields and regions around the intraparietal sulcus [corresponding to the parietal reach region (PRR) and lateral intraparietal (LIP) area in the monkey]. The interpretation of these investigations is that cortical modules encoding pointing-specific maps are largely effector independent.

This conclusion is also supported by neurophysiologic data from monkeys. The classically held view that the LIP area is essentially involved in the generation of saccadic eye movements, while the PRR is dedicated primarily to the generation of aiming arm movements, has been challenged by the finding that the LIP area and PRR contain neurons that are either responsive to both effectors or even specific for the “wrong” effector (Snyder et al., 1997; Colby and Goldberg, 1999; Gottlieb and Goldberg, 1999). One can then surmise that whenever a visual stimulus activates the saliency map in the posterior parietal cortex (PPC) and elicits an orienting eye movement, a covert coarse motor plan for the arm is also formed, even if a hand pointing movement is not required.

Furthermore, the superior colliculus (SC) deep layers contain reach-related neurons (Werner, 1993; Kutz et al., 1997; Stuphorn et al., 2000), a finding that is particularly relevant in our context considering the pivotal role of the SC in the generation of reflexive saccades, such as the ones elicited in this study. It has been demonstrated that SC reach-related neurons fire shortly before and during arm reaches with a specific movement vector, i.e., with a particular direction and amplitude (Werner, 1993; Stuphorn et al., 2000); these neurons present a high correlation between their firing and the EMG activity pattern of shoulder muscles (Werner et al., 1997; Stuphorn et al., 1999). For 40% of SC reach-related neurons, the movement vector is coded in a retinal reference frame, i.e., they are locked to a gaze-related coordinate system (Stuphorn et al., 2000). These data have been interpreted as demonstrating a role of the SC in eye-hand coordination (Lünenburger et al., 2001). Consistent with these electrophysiological studies in the monkey, recent neuroimaging investigations provide evidence for reach-related neurons also in the human SC, both in deep and superficial layers (Linzenbold and Himmelbach, 2012; Himmelbach et al., 2013).

The PPC and the SC are heavily connected by anatomical projections and are well-known to play a crucial role in the generation of reflexive saccades. The current study demonstrated that visually guided saccades are accompanied by changes in CSS excitability compatible with a pointing hand movement toward the gaze target. Therefore, the presence of reach-related neurons in both neuronal maps, in register with gaze-related neurons, constitutes an important neurophysiological correlate for interpreting the main finding of this paper.

### Conflict of interest statement

The authors declare that the research was conducted in the absence of any commercial or financial relationships that could be construed as a potential conflict of interest.
